# Peer Review of “Detecting Substance Use Disorder Using Social Media Data and the Dark Web: Time- and Knowledge-Aware Study”

**DOI:** 10.2196/58321

**Published:** 2024-05-01

**Authors:** Anonymous

**Keywords:** opioid, substance use, substance use disorder, social media, US, opioid crisis, mental health, substance misuse, crypto, dark web, users, user perception, fentanyl, synthetic opioids, United States

*This is the peer-review report for “Detecting Substance Use Disorder Using Social Media Data and the Dark Web: Time- and Knowledge-Aware Study.”*

## Round 1 Review

### Comments for Authors

1. The paper [1] is well written and easy to understand. See comments below for a summary description of the paper from my perspective.
2. However, I would have liked to see insights ideally established in the medical literature and supported by the experimental context in this paper (eg, those that can substantiate the prediction results and how this type of artificial intelligence can benefit substance use disorder [SUD]–related outcomes).
3. Although a temporal pattern–aware method is implemented in this paper, which is a big positive, I would like to see an analysis over two distinctly separate time periods to establish the consistency and robustness of the proposed approach.
4. Without addressing points 2 and 3, the utility of this work is fairly limited. I would suggest a detailed discussion of points 2 and 3 in a revised version of the paper before submission.

### Paper Summary

This paper presents a novel approach to SUD from social media posts crawled from various dark web sources. The pipeline is sufficiently novel and high-performing compared to the presented baselines and generally in isolation (80% plus is a good score). The authors specify the intended outcome of the study as establishing a relationship between the mention of drugs in posts versus SUD by analysis of the form of expression. The methodology, successes, and failures in detection are clearly stated and discussed.

## Abbreviations

SUD: substance use disorder
